# Role of *L. plantarum* KX519413 as Probiotic and Acacia Gum as Prebiotic in Gastrointestinal Tract Strengthening

**DOI:** 10.3390/microorganisms7120659

**Published:** 2019-12-06

**Authors:** Honey Chandran Chundakkattumalayil, Sreelekshmi Kumar, Rakhie Narayanan, Keerthi Thalakattil Raghavan

**Affiliations:** School of Biosciences, Mahatma Gandhi University, Kottayam 686560, Kerala, India; honeychandra88@gmail.com (H.C.C.); sreeoachira@gmail.com (S.K.); nrakhie@gmail.com (R.N.)

**Keywords:** probiotic, prebiotic, synbiotic, *Lactobacillus plantarum*, acacia gum, TNF-α

## Abstract

Probiotics, prebiotics, and synbiotics are potential mediators to maintaining healthy intestinal flora and have garnered an area of wide research in the past few years. The current study assesses the in vivo effects of probiotic (*Lactobacillus plantarum* MBTU-HK1), prebiotic (acacia gum) (either singly or in combination as a synbiotic on growth performance), biochemical, hematological, physiological, and immunological effects and their role in the reduction of procarcinogen enzyme activities in male Balb/c mice. The absence of treatment-related toxicity and a normal physiological range of biochemical and hematological parameters ensure their safe consumption. The synbiotic group was found to possess lowered cholesterol levels and enhanced protein and mineral content. The probiotic and synbiotic groups reinforced immunoglobulin levels and had a modulatory effect on phagocytosis. A lymphocyte proliferation pattern suggested the stimulatory effect of synbiotic combination on splenocyte viability and proliferation. Total antioxidant capability in the liver was determined by a 2,2-diphenyl-1-picrylhydrazyl assay and all the treatment groups were found to possess increased scavenging activity. Synbiotic and prebiotic treatment was observed to lead to reduced tumor necrosis factor α (TNF-α) levels. Bacterial procarcinogenic fecal enzyme activities were found to be decreased, proving their role in the prevention of colon cancer incidence. This study proves the potency and safety of oral administration of *L. plantarum* MBTU-HK1 and acacia gum either individually or in combination.

## 1. Introduction

“Let food be thy medicine and medicine be thy food”, as stated by Hippocrates, is the thought of today’s health-aware population. The Russian Nobel prize winner, Eli Metchnikoff, observed the beneficial role of bacteria within the gastrointestinal tract of humans [1]. The term “probiotic” comes from two Greek words (“pro” and “bios”) and means “for life” [2]. Probiotics are “a live microbial feed supplement which beneficially affects the host animal by improving its intestinal microbial balance” [3]. *Lactobacillus plantarum* is generally used as a probiotic and is “generally recognized as safe” (GRAS), has a qualified presumption of safety (QPS) status, has a high ability to survive in the gastrointestinal tract (GI) and adhere to its epithelial cells, and most importantly is a safe strain (Food and Agriculture Organization and World Health Organization) for animals and humans [4]. Prebiotics (non-digestible food ingredients) help the survival of probiotic strains. The identification of a prebiotic that confers synergistic effects with a probiotic is, thus, of great commercial value [5]. Acacia gum is a soluble fiber used as a food additive; it comes under the GRAS classification and also functions as a prebiotic [6,7]. Probiotics along with prebiotics presented in a product are called synbiotics. In 1995, Gibson and Roberfroid introduced the term “synbiotic” to describe the union between probiotics and prebiotics synergistically acting on health when compared to the action of the probiotic or prebiotic alone [8]. It is well known that oral administration of probiotics has shown promising results in experimental animal models [9]. Consumption probiotics cause the diminution of many illnesses, including cardiovascular disease, cancer, and diarrhea, etc. The modification of both the composition and metabolic activity of intestinal flora is believed to be the basis for the nutritional benefits of probiotics [5]. The present study evaluates the in vivo effect of probiotics, prebiotics, and synbiotics in Balb/c mice following oral administration. To date, though, there has been little study of *L. plantarum* MBTU-HK1 (isolated from a honey bee gut) and acacia gum, and there is no scientific evidence on the in vivo probiotic properties of *L. plantarum* MBTU-HK1 from *Apis cerana indica*. Thus, we evaluated the effect of oral administration of probiotics (*L. plantarum* MBTU-HK1), synbiotics (*L. plantarum* MBTU-HK1 and acacia gum), and prebiotics (acacia gum) on general health and histological, hematological, and immunological parameters and pro-carcinogenic bacterial enzymes in mice. 

## 2. Results

### 2.1. Evaluation of the Effect of Oral Administration of Probiotics/Prebiotics/Sybiotic on Balb/c Mice

#### 2.1.1. Persistence Studies

**Figure 1:**
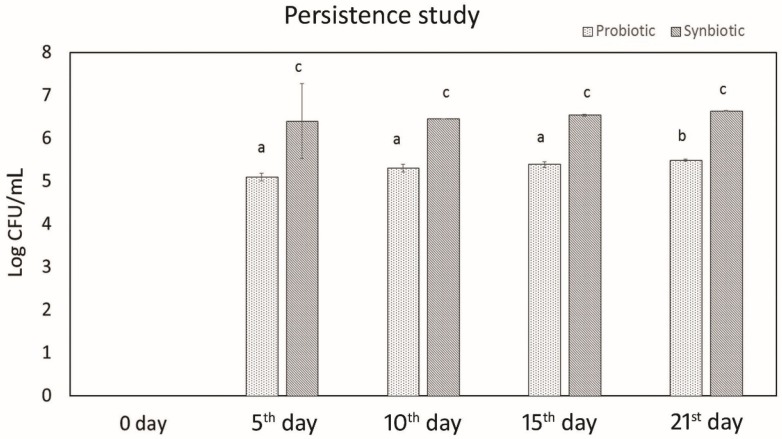
Persistence of probiotics and synbiotics in Balb/c mice. The data are expressed as mean ± standard error of the mean (SEM) (n = 6). Values with different superscripts are significantly different, where *p* < 0.05. Legend: CFU, colony forming units.

Colonies of probiotic *L. plantarum* MBTU-HK1 appeared in the fecal sample after 3 days of oral administration (Figure 1). With each 5 days of treatment, the colonization increased. The synbiotic group (*L. plantarum* MBTU-HK1 and acacia gum treated) exhibited higher counts than that of the probiotic group (which was only *L. plantarum* MBTU-HK1 treated).

#### 2.1.2. Measurements of General Health

**Figure 2:**
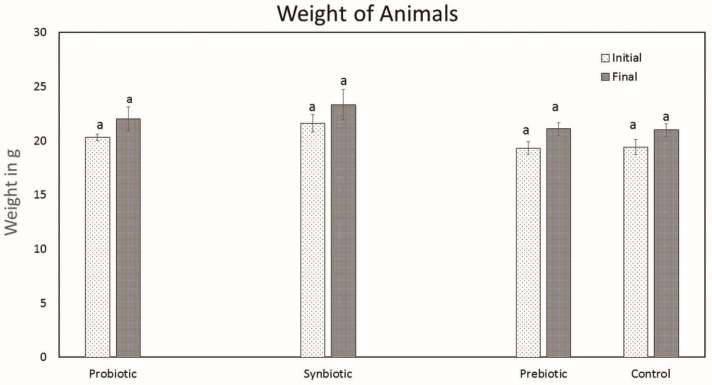
Measurement of weights (g) after 3 weeks of probiotic, synbiotic, and prebiotic treatment (n = 6). Data are expressed as mean ± SEM. Values with different superscripts are significantly different, where *p* < 0.05.

There was no change in general health appearance between the treatment and control groups. Oral administration of probiotics, synbiotics, and prebiotics for the treated groups had no adverse effects on the mice (no mortality, abnormal activity, change in food and water intake or symptoms of illness were observed). There was no significant change in the weight of animals between the test groups and control groups after 3 weeks of treatment (Figure 2). 

#### 2.1.3. Analysis of Gastrointestinal Tract Colonization 

**Figure 3:**
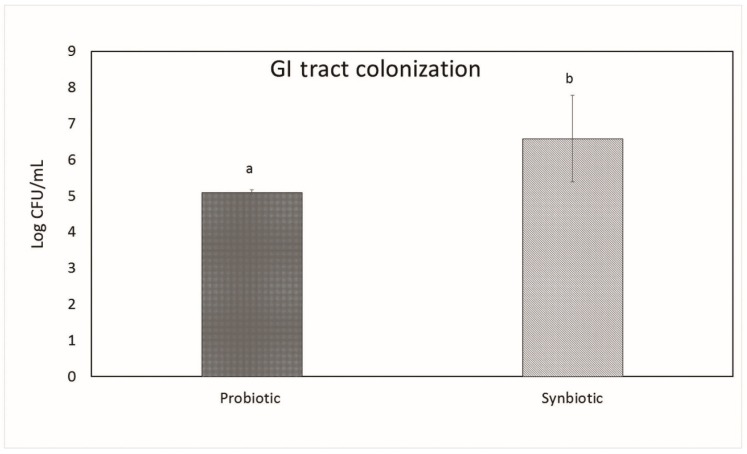
Gastrointestinal (GI) tract colonization of probiotics and synbiotics in Balb/c mice after 3 weeks of treatment. The data are expressed as mean ± SEM (n = 6). Values with different superscripts are significantly different, where *p* < 0.05.

A considerable number of colonies of *L. plantarum* MBTU-HK1 were obtained in the intestinal samples after 3 weeks of treatment, confirming the ability of *L. plantarum* MBTU-HK1 to colonize the gastrointestinal epithelial wall of mice (Figure 3). A significant increase in the number of colonies was observed in groups treated with synbiotics (i.e., both *L. plantarum* MBTU-HK1 and acacia gum) compared to groups treated with probiotics (i.e., only *L. plantarum* MBTU-HK1). 

#### 2.1.4. Bacterial Translocation

**Figure 4:**
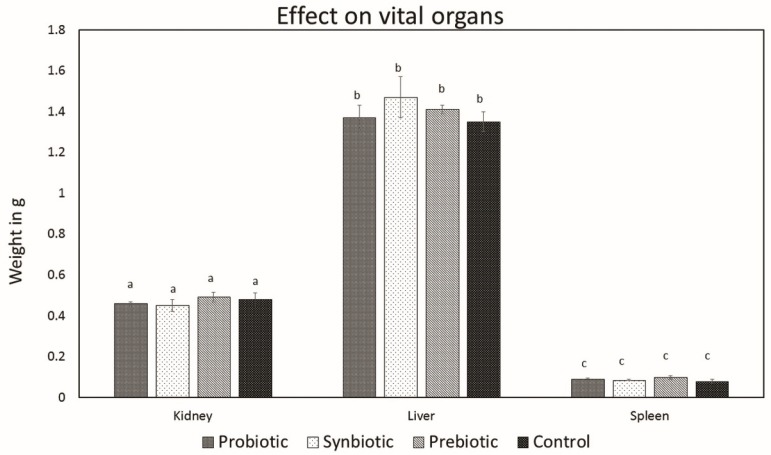
Effect of oral administration of probiotics, synbiotics, and prebiotics on the weight of the vital organs of the test and control groups. Data are expressed as mean ± SEM (n = 6). Values with different superscripts are significantly different, where *p* < 0.05.

On culturing the vital organs (kidneys, liver, and spleen) after 3 weeks of treatment, there was no growth of *L. plantarum* MBTU-HK1 on the MRS agar plates, indicating the absence of translocation of *L. plantarum* MBTU-HK1 from the gastrointestinal tract to other organs. Additionally, there was no significant change in the weight of vital organs representing the absence of splenomegaly or hepatomegaly (Figure 4).

#### 2.1.5. Histopathological Study

**Figure 5:**
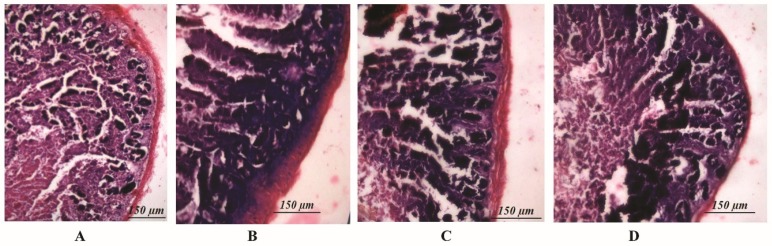
Histopathological study of intestinal sections of mice which received (**A**). probiotics only, (**B**) synbiotics, (**C**) prebiotics only, and (**D**) none of the above (control group).

Evaluation of tissue sections belonging to the probiotic, synbiotic, and prebiotic groups and the control group led to the following observations: there were no symptoms of infection, inflammation, or epithelial erosion. Thickness of the mucosal layer was unaffected, indicating an absence of infection after oral administration of the potential probiotic (Figure 5).

#### 2.1.6. Hematological Parameters of Balb/c Mice

Hematological analysis after 3 weeks of treatment revealed that the synbiotic and probiotic groups possessed significant enhancement of Hb content compared with the control and that a non-significant change could be observed between the prebiotic group and the control. On evaluation of total count, MCV, and MCH, there was no significant variation in the treatment groups from the control groups. The RBC count was significantly increased in the probiotic and synbiotic groups compared to the control. However, the prebiotic group did not possess any significant enhancement in RBC count (Table 1).

#### 2.1.7. Determination of Biochemical Parameters

The level of cholesterol was significantly reduced in both the probiotic and synbiotic groups in comparison with the control group and a non-significant reduction was observed in the prebiotic group. Among the treatment groups, the synbiotic group was found to have the lowest cholesterol level of 100.7 ± 1.7 mg/dL. When considering the levels of triglycerides, all treatment groups possessed significantly reduced levels compared to the control. The synbiotic group exhibited the lowest triglyceride level of 110 ± 4.9 mg/dL. For HDL, none of the treatment groups showed significant changes from the control group. The level of LDL was significantly reduced in all the treatment groups compared to the control, and the prebiotic group had the lowest level of LDL, 47.8 ± 0.1 mg/dL. In the probiotic and synbiotic groups, the total protein level was significantly increased, while the prebiotic group showed no significant change compared with control. Results for the level of calcium and phosphorous for all treatment groups were significantly increased compared with the control group. The highest calcium level was observed for the probiotic group, which had a level of 9.17 ± 0.035 mg/dL, while the synbiotic group had a maximum level of 9.65 ± 0.02 mg/dL of phosphorous. Table 2 shows the biochemical analysis of all the treatment groups. 

#### 2.1.8. Immunomodulatory Effect in Balb/c Mice

##### Humoral Immune Response

Table 3 shows the level of immunoglobulins in different groups. After 3 weeks of treatment, the probiotic and synbiotic group possessed increased levels of Ig G (*p* ˂ 0.05) when compared to the control group. There was a non-significant change in the level of Ig M for the control as well as the treatment groups. The level of Ig A was significantly increased in the probiotic group, reduced in the prebiotic group, and not significantly changed in the synbiotic group. Secretory Ig A was significantly enhanced in the probiotic and synbiotic groups and reduced in the prebiotic group compared with that in the control group.

##### Cell-Mediated Immune Responses

**Figure 6:**
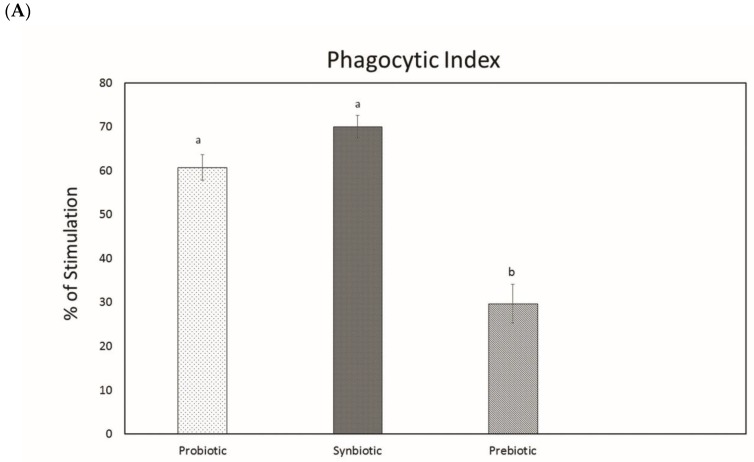

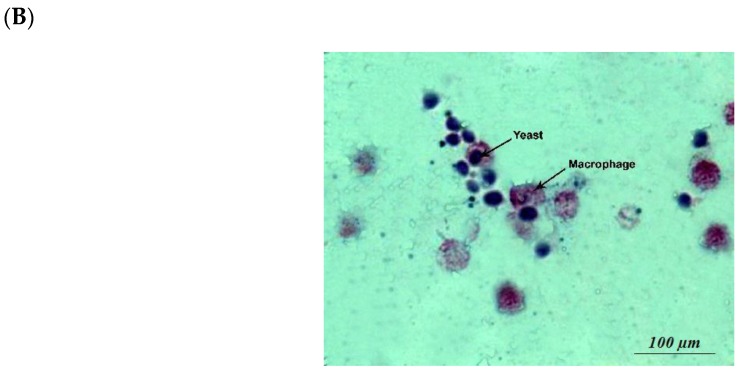
Determination of Phagocytosis. (**A**) Phagocytic activity of peritoneal macrophages of mice. Data are expressed as mean ± SEM (n = 6). Values with different superscripts are significantly different, where *p* < 0.05. (**B**) Engulfment of yeast cells by peritoneal macrophage.

A significant increase in the phagocytic activity of the peritoneal macrophages was detected in the probiotic (60.6 ± 2.9%) and synbiotic groups (70 ± 2.5%) compared with the prebiotic group (29.6 ± 4.41%). The results are given in Figure 6A,B. 

**Figure 7:**
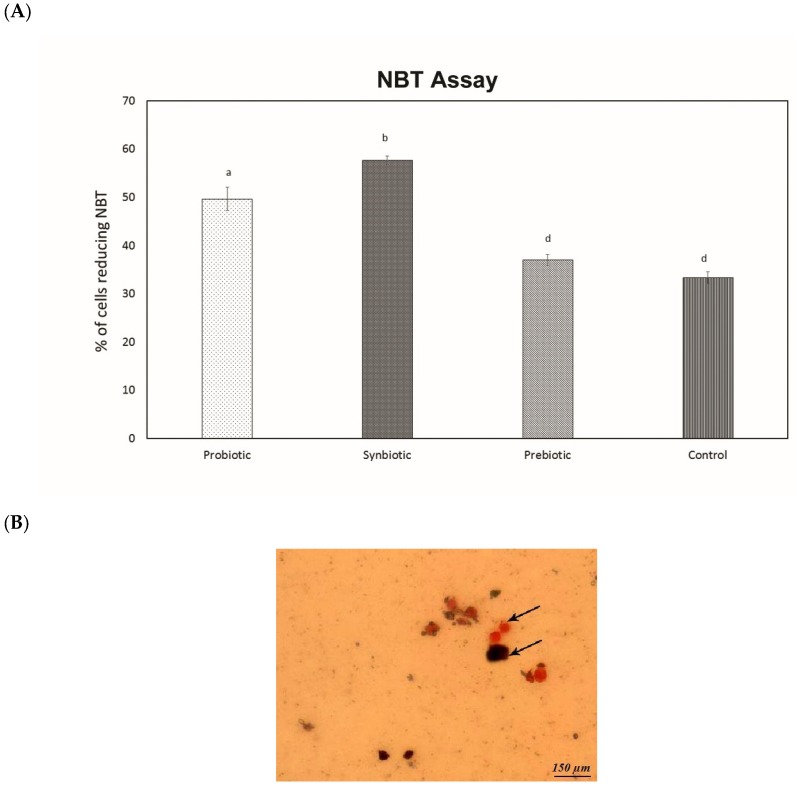
Enhancement of Phagocytosis. (**A**) Nitroblue tetrazolium (NBT) assay. Data are expressed as mean ± SEM (n = 6). Values with different superscripts are significantly different, where *p* < 0.0001. (**B**) Respiratory burst by macrophages (in black).

The enhancement of phagocytosis by the treatment group was further verified by the NBT assay. The percentage of cells reducing the NBT (electron accepter) was used as a measure of the respiratory burst exhibited by the macrophages. Significant enhancement of the number of cells reducing NBT was observed in the probiotic group (49.6%) and the synbiotic group (57.6%). Among the two groups, the synbiotic group was found to reduce NBT more efficiently. The results are depicted in Figure 7A,B. 

##### Splenic Lymphocyte Proliferation 

**Figure 8:**
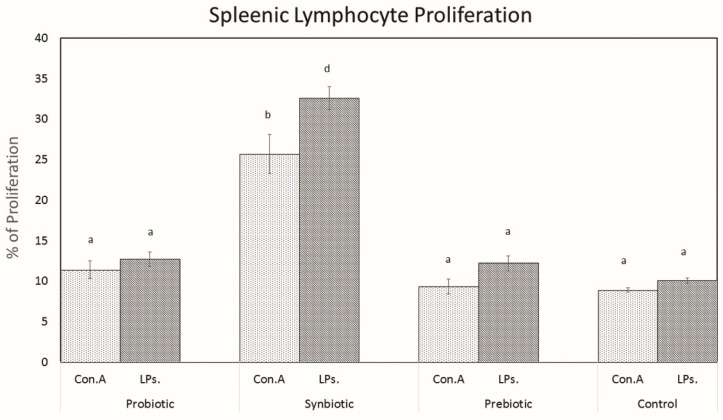
Splenocyte proliferation after lipopolysaccharide (LP) and con A stimulation. Data are expressed as mean ± SEM (n = 6). Values with different superscripts are significantly different, where *p* < 0.0001.

The potential of probiotics, synbiotics, or prebiotics to induce proliferation of splenocytes was evaluated by an MTT assay using mitogens concanavalin A and lipopolysaccharide. Figure 8 shows the proliferation of splenocytes after stimulation with con A and lipopolysaccharides (LPs). Among the two mitogens, the proliferative response was greater towards the LPs (B cell mitogen) than con A (T cell mitogen). The synbiotic group possessed 32.5% of the proliferation when using the mitogen LPs and possessed 25.6% of the proliferation when using con A as a mitogen. Only the synbiotic group exhibited a significant increase in proliferation when compared with the control group (Figure 8). 

#### 2.1.9. Antioxidant Status of Liver

**Figure 9:**
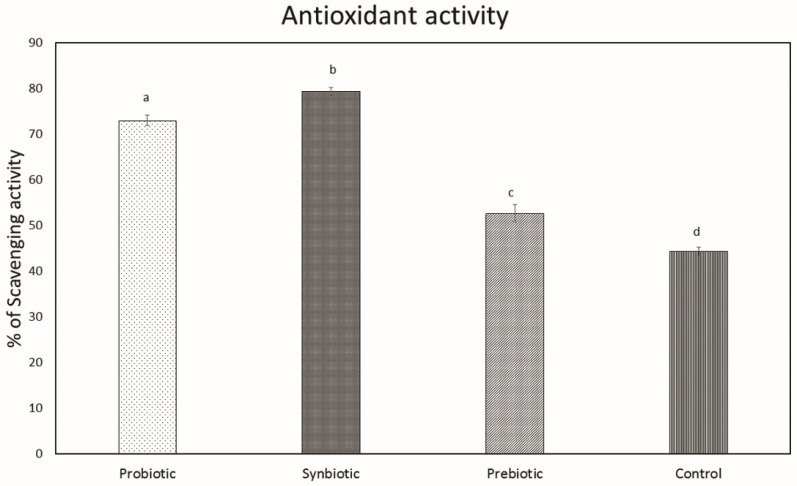
Scavenging activity of liver tissues of mice obtained using DPPH. Data are expressed as mean ± SEM (n = 6). Values with different superscripts are significantly different, where *p* < 0.0001.

The influence of treatment on the antioxidant properties of the liver was evaluated using DPPH (2,2-diphenyl-1-picrylhydrazyl) radicals. The scavenging activity of liver tissues of all treatment groups was significantly increased compared with that of the control. Among the treatment groups, the synbiotic group (79.3%) possessed the highest scavenging activity, followed by the probiotic group (73%); the prebiotic group (52.7%) was found to have the least scavenging activity (Figure 9).

#### 2.1.10. TNF-α Production

**Figure 10:**
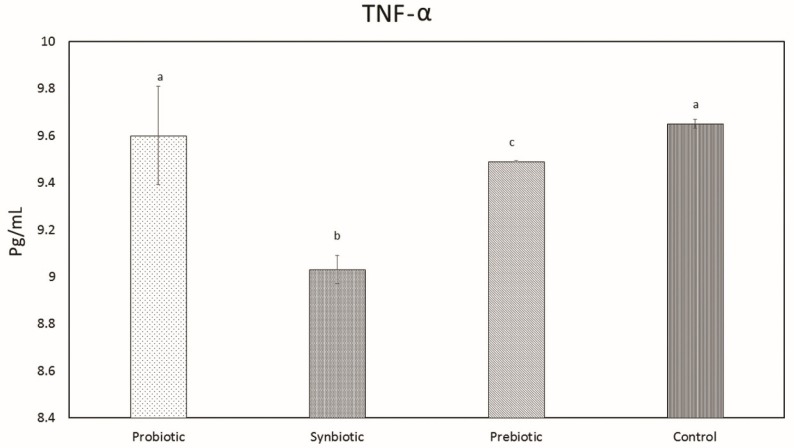
TNF-α levels in serum of mice after 3 weeks of treatment. Data are expressed as mean ± SEM (n = 6). Values with different superscripts are significantly different, where *p* < 0.0001.

The levels of proinflammatory cytokine TNF- α were significantly reduced in the synbiotic group and the prebiotic group (9.49 pg/mL) compared with the probiotic group (9.6 pg/mL) and the control group (9.65 pg/mL) (Figure 10). The synbiotic group possessed the lowest level of TNF-α (9.03 pg/mL).

#### 2.1.11. Assessment of Procarcinogenic Bacterial Enzyme Assay

##### Determination of β-Glucuronidase Activity

**Figure 11:**
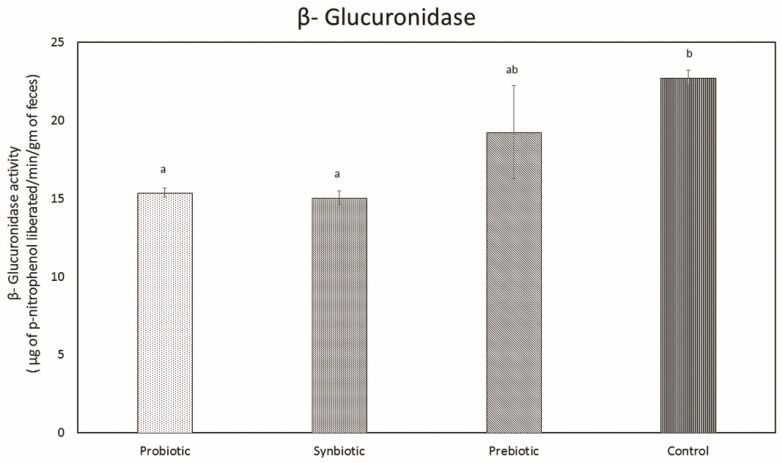
β-glucuronidase activity in the feces of the control and experimental groups. Data are expressed as mean ± SEM (n = 6). Values with different superscripts are significantly different, where *p* < 0.05.

The level of β-glucuronidase activity was significantly reduced in the probiotic and synbiotic groups and saw no change with the prebiotic group. The probiotic and synbiotic groups possessed β-glucuronidase activity levels of 15.38 ± 0.24 and 15.03 ± 0.43, respectively. Figure 11 shows the β-glucuronidase activity of the feces samples of the control and experimental groups. 

##### Determination of β-Glucosidase Activity

**Figure 12:**
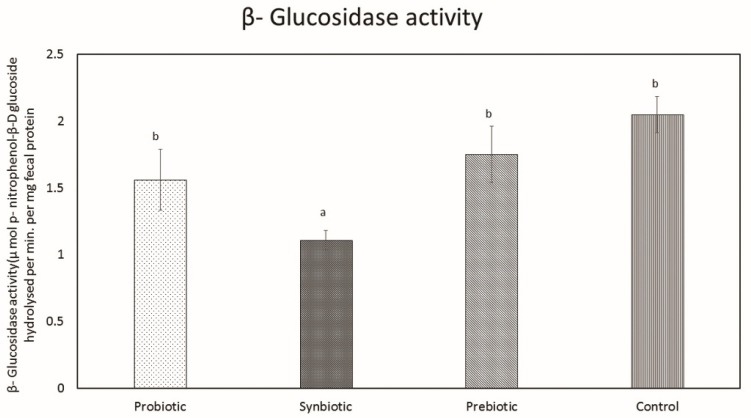
β-glucosidase activity in the feces samples of the control and experimental groups. Data are expressed as mean ± SEM (n = 6). Values with different superscripts are significantly different, where *p* < 0.05.

Levels of fecal enzyme β-glucosidase in the experimental and control groups are presented in Figure 12. In all the experimental groups, a significant reduction was observed only with the synbiotic group, and no effect was observed on administration of probiotics or prebiotics separately. The level of β-glucosidase activity detected in the synbiotic group was 1.1 ± 0.07. 

## 3. Discussion

*L. plantarum* MBTU-HK1 (accession no. KX519413) has been previously reported to have potent probiotic capabilities [10]. The aim of the current study was to evaluate the in vivo effects of probiotic *L. plantarum* MBTU-HK1 and prebiotic acacia gum in Balb/c mice. This is believed to be the first report which has proven the in vivo effects of probiotics from a honey bee gut and their synbiotic role with acacia gum. Increasing health consciousness and high rates of lifestyle diseases make our study significant for both the neutraceutical and pharmaceutical fields. In accordance with WHO guidelines, in vivo studies are obligatory for confirmation of in vitro results of probiotics and prebiotics [11]. The animal model used in this study was male Balb/c mice, a mammalian animal model which meets a taxonomic equivalency towards humans and is similar to human physiology [12]. The animals in the probiotic group were given 1 mL (containing 10^8^ viable cells) of the test strain *L. plantarum* MBTU-HK1, those in the prebiotic group were given 1% acacia gum, and those in the synbiotic group received both *L. plantarum* MBTU-HK1 (10^8^) and acacia gum (1%) orally for 3 weeks. The control group received 1 mL of normal saline. In order for the probiotic effects of *L. plantarum* MBTU-HK1 to be observed, *L. plantarum* MBTU-HK1 should harbor in the GIT. The capability of the test strains to colonize the gastrointestinal tract of Balb/c mice and the influence of acacia gum on colonisation was observed by periodic examination of serially diluted feces collected from the animals. Colonies of the test strain observed on MRS plates from the third day of treatment and the number of colonies typical to the test strain increased with the number of days of treatment. The synbiotic group (*L. plantarum* MBTU-HK1 and acacia gum treated) exhibited higher counts of probiotic *L. plantarum* MBTU-HK1 than the probiotic group (without acacia gum), suggesting the prebiotic potency of acacia gum on *L. plantarum* MBTU-HK1. Results in [13] have proposed that 1% acacia gum promotes the growth of lactobacilli more efficiently than a 2% dosage, thereby meaning it can exert potential benefits to the host. Colonization and survivability in the digestive tract are measured as the prime criteria for the effective functioning of the health-promoting role of probiotics [2]. The in vitro adhesion capability of the test strain [10] was further confirmed by culturing matter of the intestine as soon as the animals had been sacrificed. Efficient colonization of the gastrointestinal tract by *L. plantarum* MBTU-HK1 is in agreement with the results of [14], in which the *L. plantarum* strain ULAG24 exhibited favorable adherence to gut epithelia cells. According to [15] the establishment of an introduced strain not only depends on the route of administration but also on the interaction of microorganisms with the GI tract of host organisms. Advantageous probiotic effects can by attained by the colonisation by probiotic microorganisms of the intestinal surface mucus layer. This makes them affect the intestinal immune system, displace enteric pathogens, and provide antioxidants and anti-mutagens and possibly other effects by cell signaling [16]. Results of the current study indicated that there was no adverse effect on the general health status of the mice belonging to both the treatment and control groups with the oral administration of probiotics (*L. plantarum* MBTU-HK1), synbiotics (*L. plantarum* MBTU-HK1 and acacia gum) and prebiotics (acacia gum). Throughout the experimental periods, we did not observe any behavioral changes, changes in general health appearance, weight loss, diarrhoea, or any other adverse clinical signs, and we also did not see signs of hepatomegaly or splenomegaly as reported in [17,18]. Histopathological evaluation of the gastrointestinal tracts of animals belonging to the treatment groups did not produce any signs of inflammation, abscesses, epithelial erosion, or mucosal thickening, indicating the safety of the test strain (*L. plantarum* MBTU-HK1) and acacia gum for use as a probiotic and prebiotic, respectively, which is in agreement with the findings of [19,20]. Based on the hematological parameters, all the treatment groups possessed better health status on oral administration of *L. plantarum* MBTU-HK1 and acacia gum separately and in combination. There was a usual increase in the counts of Hb and RBCs in mice administered with *L. plantarum* MBTU-HK1 and acacia gum (i.e., in the probiotic group and the synbiotic group) compared with the control. [21] has reported that there was a significant increase in RBCs in an *L. plantarum* IS-10506-supplemented animal group, which plays a role in tissue oxygen delivery. Experiments in [22] concluded that the degradation of acacia gum brings about short chain fatty acids, which promote fetal hemoglobin expression in RBCs. There is a direct association between RBC and Hb concentration by which a reduction in one parameter produces a reduction in another parameter [23]. Enhanced levels of Hb and RBC are considered a sign of a non-anemic condition. Our results are in agreement with the observations of [19,24]. The WBC count and MCV and MCH levels of animals administered with the test strain and acacia gum were not significantly different from those of the control group, with values of all the parameters residing within the normal range. Similar reports were observed in [19], which found that the administration of *Lactobacillus rhamnosus* HN001, *Lb. acidophilus* HN017, and *Bifidobacterium lactis* HN019 had no effect on WBC count and MCV and MCH levels. 

The energy profile and mineral profile of the experimental animals were evaluated to analyze the effects of oral administration of *L. plantarum* MBTU-HK1 as a probiotic and acacia gum as a prebiotic. The level of cholesterol was significantly reduced in the probiotic group and the synbiotic group (which contained acacia gum as a prebiotic). It is known from our previous in vitro studies that *L. plantarum* MBTU-HK1 has the ability to assimilate cholesterol from growth media (due to the production of the BSH enzyme), and thus the reduced level of cholesterol in the probiotic and synbiotic groups can be attributed to the bile salt hydrolase activity of the test strain. The results obtained in the in vitro study have thus been proven in the in vivo experiments in [25,26,27,28]. In all the treatment groups triglycerides were reduced and the synbiotic group possessed the lowest level of triglycerides, which is supported by the results of [29], which demonstrated that *L. acidophilus* and *B. bifidum* can significantly decrease the serum levels of LDL and triglycerides. Ref. [30] discovered the novelty of acacia gum in decreasing the level of LDL and triglycerides, thus showing that they play a role in modulating the lipid profile of patients associated with SCD and dyslipidema. When considering the levels of HDL and LDL, all the treatment groups exhibited insignificant changes compared to the control. The mineral profile of all the treatment groups showed increased levels of inorganic phosphorus and calcium compared to those of the control group. According to [31], probiotic supplementation can help to digest and absorb more calcium and supports bone metabolism, resulting in higher bone density and strength in birds. Probiotics produce short chain fatty acids which increase the solubility of available calcium and simultaneously decrease para-thyroid hormone (PTH) levels (increased PTH levels cause bone resorption by stimulating the osteoclasts), minimizing bone loss [32]. Oral administration of probiotics and synbiotics significantly enhanced the levels of total protein. Ref. [33] observed that the total protein level was higher with supplementation of *L. plantarum*, and that proteolytic enzymes produced by the strain enhanced the digestion and assimilation of dietary protein. There was no significant difference in the total protein of the prebiotic group at 3 weeks of treatment, and thus acacia gum had no effect on the total protein level. This result is in agreement with the findings of [34]. 

The amounts of serum Ig M were unaffected by the administration of probiotics, synbiotics, and prebiotics, though the level of serum Ig G was significantly enhanced in the synbiotic group and the level of Ig G was increased upon colonization with intestinal bacteria [35]. After three weeks of colonization an improved level of serum Ig A was observed in the probiotic group administered with *L. plantarum* MBTU-HK1. Serum Ig A had an anti-inflammatory effect by regulating the deliverance of inflammatory mediators such as TNF-α and IL-6 [35]. The levels of secretory Ig A were increased in the probiotic and synbiotic groups and the highest level was found in the probiotic group. These results are supported by the observations of [36], who concluded that oral administration of *L. plantarum* AYA in mice caused an increase in Ig A production in the small intestine and provided protection against respiratory influenza virus infection. Secretory Ig A (sIg A) functions to maintain local homeostasis and sIg A antibodies bind to commensal and pathogen bacteria and toxins, blocking them via immune exclusion [37]. When considering the immunoglobulin levels of the prebiotic group, acacia gum had no effect on serum Ig G and Ig M but reduced Ig A and sIg A levels, which is supported by the results of [38].

Phagocytosis is considered an initial vital phenomenon in host defense against the infected pathogen. As per our results, both the probiotic and synbiotic groups possessed significantly enhanced phagocytic activity compared to the prebiotic group, indicating their ability to boost up the immune system [39]. Experiments in [40] have suggested that the administration of *Lactobacillus plantarum* may have a positive effect on the immune response in immunocompromized hosts. Following this, the consumption of probiotics and interaction of the bacteria with intestinal enterocytes initiate a host response, since intestinal cells produce various immunomodulatory molecules when stimulated by the bacteria. Ref. [41] has reported that *Lactobacillus plantarum* is able to produce exoploysaccharide, which may be a factor that enhances the ability of *Lactobacillus plantarum* to induce phagocytosis; our previous study [10] has proven the ability of the test strain to produce exopolysaccharides. During phagocytosis, a metabolic process known as the respiratory burst occurs in activated macrophages. This process results in the activation of this membrane-bound oxidase that catalyzes the reduction of oxygen to a superoxide anion, which is a reactive oxygen intermediate extremely toxic to pathogens. In this regard, the synbiotic group possessed a strong ability to induce respiratory burst, followed by the probiotic and prebiotic groups when compared with the control group. Our study suggests the ability of the test strain to reinforce the immune response of phagocytes. Increased respiratory burst activity can be correlated with increased bacterial pathogen killing activity of phagocytes [42]. 

The effect of probiotic supplementation on splenic lymphocyte proliferation has been assessed by many investigators to analyze the effects of probiotics on immune function. The proliferative responses of spleen cells to concanavalin A (a T-cell mitogen) and lipopolysaccharides (a B-cell mitogen) in mice administered with different probiotic strains have been reported by [43]. The LPs were used as a mitogen for the proliferation of B cells, whereas con A was used for T cell proliferation [44]. In the present study we evaluated the effects of *L. plantarum* MBTU-HK1, acacia gum, and their combination on splenic lymphocyte proliferation. The maximum proliferative effect was observed only in the synbiotic group in the presence of LPs, which are involved in B cell proliferation. Ref. [45] has confirmed the enhanced splenocyte proliferation of *L. plantarum* KLDS1.0318.

The antioxidant properties of liver tissue collected from all the experimental groups were evaluated after 3 weeks of treatment. All treatment groups possessed significantly enhanced antioxidant activity compared with the control and the highest activity was exhibited by the synbiotic group, followed by the probiotic group and the prebiotic group. The in vitro antioxidant activity of *L. plantarum* MBTU-HK1 was proven in the in vivo study. There have been many reports regarding the antioxidant properties of strains of *L. plantarum* [46]. Exopolysaccharides (EPs) from LAB may be considered an effective non-toxic substance with antioxidant activity. EPs producing properties of the test strain, *L. plantarum* MBTU-HK1, reinforce its antioxidant properties [10]. Reports in [47] emphasize the antioxidant potential of acacia gum. The combined effect of *L. plantarum* MBTU-HK1 and acacia gum exerted great antioxidant activity in the synbiotic group and thus has a potential effect against oxidative damage. 

In the present study, we evaluated in vivo cytokine levels after mice were administered with the probiotic *L. plantarum* MBTU-HK1, the prebiotic acacia gum, and a combination of both as a synbiotic. After 3 weeks of treatment, *L. plantarum* MBTU-HK1 had no effect on TNF-α levels. Similar results have been reported in [48], in which *L. plantarum* did not alter the secretion of TNF-α levels. TNF-α levels were significantly reduced in both the synbiotic and prebiotic groups. There have been many reports regarding the anti-inflammatory activity of acacia gum [49]. The fermentation of acacia gum by colonic bacteria releases butyrate. Butyrate serves as a potent anti-inflammatory agent by inhibiting NF_K_B (it mediates the transcription of inflammatory cytokines) [50]. The improved anti-inflammatory activity of the synbiotic may be due to the combinatory use of acacia gum, and the TNF-α-level-lowering effect of acacia gum make it significant in patients with Crohn’s disease and rheumatoid arthritis. 

Alteration of gut microbiota by probiotics and prebiotics, separately or in combination, exerts a positive impact on the link between the immune system and microbiota, having a beneficial role in inhibiting inflammation and colorectal cancer [51]. Currently, we pay much attention towards the role of two pro-carcinogenic fecal enzymes: β-glucuronidase and β-glucosidas; the more enzyme activity there is, the more free carcinogens with the potential to produce tumors will be present in the intestine [52,53]. This is why activities aimed at reduction of the enzymes’ levels are one of the main research directions for the aforementioned disease [53]. In the present study, we appraised enzyme activity in the stool samples of mice after 3 weeks of administration of *L. plantarum* MBTU-HK1 and acacia gum, separately or in combination, and the levels of β-glucuronidase were found to be significantly reduced in both the probiotic and synbiotic groups compared to the control group. While β-glucosidase activity was significantly reduced only in the synbiotic group, consumption of prebiotics and probiotics have been shown to decrease the activity of β-glucuronidase and glucosidase [54]. Normal intestinal flora have an impact on carcinogenesis by producing enzymes (β- glucosidase, β-glucuronidase, azoreductase, and nitroreductase), catalyzing the conversion of precarcinogens into active carcinogens. Probiotics may defend the host from this activity. There have been reports regarding the supplementation of *L. acidophilus* and *L. casei* in humans which have facilitated decreased levels of these enzymes [55]. Ref. [56] has studied the effect of *L. acidophilus* in reducing the activity of carcinogen-releasing bacterial enzymes (β-glucoronidase, nitroreductase, and azoreductase).

## 4. Materials and Methods

A bacterial strain, *Lactobacillus plantarum* MBTU-HK1, isolated from the gut of *Apis cerana indica* (accession no. KX519413), was selected for in vivo study. This strain has been previously reported to have potent probiotic capabilities and efficient cell surface properties [10]. *Lactobacillus plantarum* MBTU-HK1 was cultured in MRS (de Man, Rogosa and Sharpe) medium for 24–48 h at 37 °C, centrifuged at 8000 rpm for 10 min at 4 °C, and washed as a pellet with sterile phosphate buffered saline (PBS). Approximately 10^8^ colony forming units (CFU)/mL of MBTU-HK1 was resuspended in 1 mL of sterile PBS.

Acacia gum was found to be a more effective prebiotic for the growth of *Lactobacillus plantarum* MBTU-HK1. Hence, the present study selected acacia gum (1% in distilled water) as a prebiotic for in vivo analysis.

Animal experiments were approved by the Institutional Animal Ethical Committee (animal ethical clearance no. B21032014-17, 29 May 2014) and all works were performed according to the guidelines. Male 6-week-old Balb/c mice weighing 18–20 g were obtained from the Small Animal Breeding Station (SABS) at Kerala Veterinary and Animal Sciences University, Thrissur, Kerala. Animals were maintained in the animal house at the School of Biosciences, Mahatma Gandhi University, Kottayam, and Kerala. Animals were housed in cages and were fed on a standard sterile pellet diet and had sterile water ad libitum. Bedding in cages was changed daily. The animals were maintained under controlled conditions of a temperature of 26–28 °C with a 12 h light to 12 h dark cycle. The study period was 5 weeks (2 weeks acclimatization and 3 weeks treatment). After acclimatization, mice were equally distributed in four experimental groups (n = 6/group). Treatment groups for the study included three experimental groups and a single control group. For the period of experiment, behavioral changes, treatment-related illnesses or death, and change in hair luster between the control and test groups were examined. Experimental groups and oral administration were (1) the probiotic group (administered with 10^8^ CFU/mL of MBTU-HK1 by an orogastric gavage with a ball-tip needle), (2) the prebiotic group (administered with acacia gum only, 1%, in distilled water), The (3) synbiotic group (administered with 10^8^ CFU/mL of MBTU-HK1 along with 1% acacia gum) and (4) the control group (inoculated with sterile PBS only, 1 mL). The treatment was given consecutively for 21 days (3 weeks).

### 4.1. Evaluation of the Effect of Oral Administration of Probiotics/Prebiotics/Sybiotics on Balb/c Mice

#### 4.1.1. Persistence Studies

Fresh fecal pellets were collected from mice in each group separately at 3, 5, 7, 15, and 21 days during the period of treatment. 1 gm of feces was homogenized and serially diluted in PBS. Enumeration of colonies was done by serial dilution followed by the spread plate method in MRS agar plates, incubation was carried out at 37 °C for 48 h, and the colonies obtained were expressed as CFU per gram of fecal pellet. 

#### 4.1.2. Measurements of General Health

The general health appearance of the mice was observed regularly. Live weights of the mice in the experimental and control groups were recorded on a Sartorius balance throughout the experiment. Feed and water intake, behavioral changes, and treatment-related illnesses or unhealthy symptoms or death were observed during the experimental period. Individual body weights of the animals were recorded on the first day of treatment and final body weights were taken prior to sacrifice on the twenty-first day. Additionally, the weight of the internal organs, such as the liver, spleen, and kidneys, were measured at the time of sacrifice and compared with the control animals.

#### 4.1.3. Analysis of Gastrointestinal Tract Colonization 

The animals in the experimental groups were sacrificed after 21 days of treatment. The stomach, small intestine, and colon of mice were taken aseptically, enumeration of viable bacteria was performed by serial dilution followed by the spread plate method in MRS agar plates, and the colonies formed were expressed in CFU [57]. 

#### 4.1.4. Bacterial Translocation

Bacterial translocation in the liver, spleen, and kidneys was measured and compared with that of the control group after that group had been sacrificed. Livers, kidneys, and spleens were homogenized in PBS, serially diluted, and plated on MRS medium. After incubation at 37 °C for 24–48 h, the colonies grown were counted and the results expressed as CFU.

#### 4.1.5. Histopathological Study 

The colonic sections of the mice were fixed in 10% formaldehyde in pH 7.4 PBS. The fixed tissues were dissected and embedded in paraffin. Five-micrometer sections were placed on slides and stained with hematoxylin and eosin or a Gram stain. The tissue sections were observed using the Phase contrast microscope Olympus via Software Q-Capture Pro7. Photomicrographs were created with a Nikon automatic camera attached imaging software [57].

#### 4.1.6. Hematological Parameters of Balb/c Mice

Blood samples from each experimental and control group were collected in ethyl diathamine (EDTA) for the determination of hematological parameters. The red blood cell (RBC) count was determined using a Neubauer chamber and whole blood, as explained by [58].

(1) $$Total\;RBC\;count=Number\;of\;cells\;counted\times 10000\;count/mm^{3}$$

The white blood cell (WBC) count was determined using the Neubauer chamber and whole blood.

(2) $$Total\;WBC\;count=Number\;of\;cells\;counted\times 50\;count/mm^{3}$$

An Agappe diagnostic kit was used for the determination of hemoglobin (Hb) in blood according to the manufacturer’s instructions [59], i.e.,

(3) $$Hb\left(g/dl\right)=\frac{O.DT\times 60\times 0.251}{O.DS}$$

where ODT is the optical density of the test at 546 nm and ODS is the optical density of the standard at 546 nm.

Blood samples were centrifuged at 1500 rpm for 10 min at 4 °C. The RBC layer was separated and parameters such as mean corpuscular volume (MCV) and mean corpuscular hemoglobin concentration (MCHC) were detected with an automated hemoanalyzer.

#### 4.1.7. Determination of Biochemical Parameters

For separation of the serum, blood was collected from the retro orbital sinus into a 2 mL vial and allowed to stand for 2 h at room temperature. The vial was then centrifuged at 12,000× *g* for 15 min at 4 °C to separate the serum and the collected serum were stored at −20 °C for analysis. Biochemical parameters such as cholesterol, triglycerides, high-density lipoprotein (HDL), low-density lipoprotein (LDL), total protein, calcium, and phosphorous were evaluated using a semi-automated clinical chemistry analyzer. 

#### 4.1.8. Immunomodulatory Effect in Balb/c Mice

##### Humoral Immune Response

Levels of Ig A, Ig G, and Ig M in serum and the levels of Ig A in intestinal fluid were analyzed using mouse Ig A, Ig G, and Ig M ELISA quantitation kits. Assays were performed according to the manufacturer’s instructions for the quantitation kits (mouse Ig G ELISA kit catalog number: E-90G; mouse Ig M ELISA kit catalog number: E-90M and mouse Ig A ELISA kit catalog number: E-90A; ICL Laboratories, Newberg, USA). Amounts of Ig A, Ig G, and Ig M in test sera were assessed by sandwich ELISA. 

##### Cell-Mediated Immune Responses

Peritoneal macrophages were obtained from the mice by intraperitoneal injection with 5 mL of ice-cold PBS (with 3% fetal calf serum (FCS)) using a 27 g needle, with the peritonea gently massaged to dislodge any attached cells into the PBS solution. A 5 mL syringe was inserted into the peritonea and the fluid collected. The collected cell suspensions were spun at 1500 rpm for 8 min at 4 °C and the cells were resuspended in PBS. The viability of the cells was checked using the Trypan blue exclusion method [60]. 

Macrophage cell suspension (0.1 mL) was smeared onto a clean glass slide and incubated (in a moist cotton pad) for 20 min at 37 °C in 5% CO_2_ to allow the attachment of the cells to the glass slide. Then, the slides were drained with sterile Hank’s balanced salt solution (HBSS), flooded with a suspension of heat-killed yeast cells, and incubated at 37 °C for one hour in 5% CO_2_. After this, the slides were fixed in absolute ethanol for 2 min, dried, and stained with Giemsa stain. The number of yeast cells phagocytosed by each macrophage were counted using an oil immersion (100× objective). The value obtained was taken as the phagocytic index (PI). The percentage of phagocytosis (immune stimulation) [61] was calculated from the PI of the test group and that of the control group using the following equation.

(4) $$\%\;\mathrm{of}\;\mathrm{Phagocytosis}=\frac{\mathrm{PI}\left(\mathrm{test}\right)-\mathrm{PI}\left(\mathrm{control}\right)}{\mathrm{PI}\left(\mathrm{control}\right)}\times 100$$

Peritoneal cell suspension (0.1 mL) was incubated in the presence of 0.1 mL NBT for 30 min at 37 °C with 5% CO_2_. A drop of the sample was placed on a glass slide. The smear was allowed to dry and was fixed with ethanol for 3 min, then stained with saffranin for 3 min and observed under a 40× objective. The percentage of macrophages was calculated by counting 100 macrophages with formazan granules [62].

##### Splenic Lymphocyte Proliferation

Spleens were obtained from both the control and test groups of mice after euthanasia, homogenized in cold HBSS, and the clumps allowed to settle in a tube kept in ice for 20 min. Supernatants were collected and resuspended in sterile RPMI 1640 media (Himedia) with HEPES (4-(2-hydroxyethyl)-1-piperazineethanesulfonic acid) buffer (20 mM), sodium bicarbonate (24 mM), and Gentamicin (100 μg/mL). The splenocytes in the upper portion of the medium were collected and centrifuged at 1000× *g* for 5 min at 4 °C, and red blood cells were lysed via an erythrocyte lysis buffer. Cells were washed twice with culture medium to remove traces of lysis buffer and finally resuspended in 1 mL of RPMI 1640 containing 10% FCS. The viability of the immune cells was checked using trypan blue exclusion [63]. One hundred microliters of cell suspension were dispensed into 96-well flat-bottomed tissue culture plates in the presence of 10 μL mitogen, concanavalin A (1.5 μg/mL), and lipopolysaccharide (5 μg/mL), separately, to determine the differences between cell proliferation. Plates were incubated at 37 °C for 72 h under 5% CO_2_. Methyl thiazolyl tetrazolium (SRL Laborataries) was dissolved in RPMI-1640 (Himedia) at 5 mg/mL added to each well, and the plates were incubated at 37 °C for 4 h. During this period, crystals of MTT (Methyl Thiazolyl Tetrazolium) formazon formed at the bottom of each well. Seventy microliters of supernatant was removed from each well and discarded, and cell dissociation solution (20% *w/v* sodium dodecyl sulfate and 50% *v/v* dimethyl sulfoxide, pH 4.5) was added to each well and mixed thoroughly by repeat pipetting to dissolve the dark blue crystals, and incubated overnight at 37 °C. Splenic lymphocyte proliferation was obtained by determining the absorbance at 570 nm using a microplate reader (Thermofischer Scientific Varioskan flash Multimode Reader (3001-1885), Vantaa, Finland). The percentage of splenocyte proliferation was calculated using the following equation.

(5) $$Splenocyte\;Proliferation\;\left(\%\right)=\frac{OD\;sample-OD\;control}{OD\;control}\times 100$$

#### 4.1.9. Antioxidant Status of Liver

Mice livers in the control and test groups were excized, cleaned, and homogenized in 1:5 volumes of normal saline solution. The homogenate was centrifuged at 1700× *g* for 10 min at 4 °C. The supernatant was collected and DPPH radical scavenging activity was determined using the method given in [64]. A working solution of 60 µmol L^−1^ of a methanolic solution of DPPH was freshly prepared. An aliquot of 10 μL of the supernatant fractions from the liver homogenate of the control and treatment mice was added to respective wells in a 96-well microtiter plate. DPPH working solution (80 μL) was added to each of the wells. Then, the plates were incubated in the dark at 37 °C for 45 min. The scavenging activity was measured spectrophotometrically by a decrease in absorbance at 517 nm. The blank value was determined by using 1.15 g KCL L^−1^. The experiment was conducted using a positive control of ascorbic acid at a concentration of 1 mg/mL.

(6) $$\%\;\mathrm{of}\;\mathrm{Scavenging}\;\mathrm{activity}=1-\left[\frac{A517\mathrm{nmSample}}{A517\mathrm{nmBlank}}\right]\times 100$$

#### 4.1.10. Tumor Necrosis Factor α (TNF-α) Production

TNF-α production was quantified using Bio Legend’s ELISA MAX™ Deluxe kit according to the manufacturer’s protocols. 

#### 4.1.11. Assessment of Procarcinogenic Bacterial Enzyme Assay

β-glucuronidase and β-glucosidase activities were assessed using the method given in [56]. Fresh fecal samples (100 mg/mL) were dissolved in cold 0.1 M potassium phosphate buffer (pH 7.0) and homogenized. Centrifugation at 1200 g for 10 min was performed to collect the supernatant, which was used for the evaluation of β-glucuronidase and β-glucosidase activities.

##### Determination of β-Glucuronidase Activity

Fecal supernatant (0.1 mL) was mixed with the reaction mixture, 0.9 mL (0.02 M potassium phosphate buffer, 0.1 mM ethylene diamine tetra acetic acid, and 0.05 mM phenolphthalein β-glucuronide). The mixture was incubated for 60 min at 37 °C, and then 5 mL of 0.2 M glycine buffer (pH 10.4) containing 0.2 M NaCl was added. With each sample, a control sample (reaction mixture without phenolphthalein β-glucuronide) was determined for the same incubation period. The volume of phenolphthalein liberated by enzymatic action was measured spectrophotometrically at 550 nm and compared with the standard curve of phenolphthalein. Enzyme activity was expressed as micrograms of phenolphthalein liberated/min/gm feces.

##### Determination of β-Glucosidase Activity

Fecal supernatant (0.2 mL) was added to a 0.8 mL reaction mixture (0.1 M phosphate-buffered saline and 1 mM p-nitrophenyl β-pyranoside). The sample mixture was incubated for 60 min at 37 °C and 5 mL of 0.1 M NaOH was added. With each sample, a control (sample mixture without p-nitrophenyl β-pyranoside) was determined for the same incubation period. The absorption was determined at 420 nm and compared with the standard curve of p-nitrophenol. The enzyme activity was expressed as micro mol p-nitrophenol, i.e., β-D glucoside hydrolyzed per min per mg of fecal protein. 

### 4.2. Statistical Analysis

Statistical analysis of the data obtained was performed by conducting a Students t-test, and values have been expressed as mean ± standard error of the mean (SEM) values. One-way analysis of variance was performed by Tukey’s test and the significance difference was taken at *p* < 0.05. All statistical analyses were performed using the software Graph Pad Prism 5.

## 5. Conclusions

The present in vivo study has indicated the possibility of safe administration of *L. plantarum* MBTU-HK1 (as a probiotic) and acacia gum (as a prebiotic) either singly or in combination (as a synbiotic). The safety evaluation exhibited extremely favorable results for general health and biochemical, hematological, and histological parameters. The consumption of a combination of the aforementioned prebiotic and probiotic as a synbiotic was shown to exert many health-improving properties, such as reduced blood lipid levels, regulation of immune function, and also reduction of the levels of carcinogen-releasing enzymes. The maintenance of healthy gut microbiota by prebiotics and probiotics can be considered a striking route for retaining human and animal health statuses and disease prevention. 

## Figures and Tables

**Table 1:** Hematological parameters. Data are expressed as mean ± SEM (n = 6).

Parameters	Probiotic	Synbiotic	Prebiotic	Control
Hb (g/dL)	12.2 ± 0.17 ^c^	12.85 ± 0.03 ^b^	11.4 ± 0.16 ^a^	11.13 ± 0.14 ^a^
WBC count (×10^9^)	9.06 ± 0.93 ^a^	9.1 ± 0.34 ^a^	8.5 ± 0.08 ^a^	8.9 ± 0.14 ^a^
MCV (fL)	45.2 ± 2.6 ^a^	43.6 ± 2.7 ^a^	46.13 ± 0.9 ^a^	45.4 ± 1.5 ^a^
MCH (pg)	15.3 ± 0.11 ^a^	15.08 ± 0.03 ^a^	15.27 ± 0.12 ^a^	15.25 ± 0.07 ^a^
RBC count (×10^12^)	8.75 ± 0.13 ^c^	9.3 ± 0.3 ^b^	8.4 ± 0.08 ^a^	7.4 ± 0.26 ^a^

Mean values in the same row with different superscripts are significantly different, where *p* < 0.05. Legend: Hb, hemoglobin; WBC, white blood cell; MCV, mean corpuscular volume; MCH, mean corpuscular hemoglobin; RBC, red blood cell.

**Table 2:** Biochemical parameters. Data are expressed as mean ± SEM (n = 6).

Parameters	Probiotic	Synbiotic	Prebiotic	Control
Cholesterol (mg/dL)	104.9 ± 2.5 ^a^	100.7 ± 1.7 ^a^	113.3 ± 3.1 ^b^	113.6 ± 2.7 ^b^
Triglycerides (mg/dL)	124 ± 2.5 ^a^	110 ± 4.9 ^b^	138 ± 0.5 ^c^	153 ± 1.7 ^d^
HDL (mg/dL)	23.6 ± 1.2 ^a^	25 ± 0.5 ^a^	23.3 ± 0.66 ^a^	23.43 ± 3.1 ^a^
LDL (mg/dL)	54.6 ± 0.12 ^a^	49.26 ± 0.03 ^b^	47.8 ± 0.1 ^c^	57.6 ± 0.2 ^d^
Total Protein (mg/dL)	5.71 ± 0.008 ^a^	5.63 ± 0.017 ^a^	5.23 ± 0.035 ^c^	5.17 ± 0.037 ^c^
Calcium (mg/dL)	9.17 ± 0.035 ^a^	8.6 ± 0.31 ^a^	8.67 ± 0.035 ^a^	7.7 ± 0.006 ^b^
Phosphorous (mg/dL)	9.6 ± 0.21 ^a^	9.65 ± 0.02 ^a^	9.49 ± 0.003 ^a^	9.03 ± 0.06 ^b^

Mean values in the same row with different superscripts are significantly different, where *p* < 0.05. Legend: HDL, high-density lipoprotein; LDL, low-density lipoprotein.

**Table 3:** Effects of probiotics, synbiotics, and prebiotics on humoral immune response.

Immunoglobulin	Probiotic	Synbiotic	Prebiotic	Control
Ig G (g/L)	1.7 ± 0.01 ^ab^	1.77 ± 0.008 ^a^	1.6 ± 0.008 ^b^	1.68 ± 0.02 ^b^
Ig M (g/L)	1.04 ± 0.09 ^a^	0.84 ± 0.02 ^a^	0.91 ± 0.008 ^a^	1.06 ± 0.03 ^a^
Ig A (g/L)	2.49 ± 0.008 ^a^	1.91 ± 0.01 ^b^	0.85 ± 0.03 ^c^	1.97 ± 0.03 ^b^
Secretory Ig A (g/L)	3.14 ± 0.026 ^a^	2.72 ± 0.021 ^b^	1.56 ± 0.12 ^c^	2.1 ± 0.057 ^d^

Mean values in the same row with different superscripts are significantly different, where *p* < 0.05. Data are expressed as mean ± SEM (n = 6).
